# Activation of the Ventrolateral Preoptic Neurons Projecting to the Perifornical-Hypothalamic Area Promotes Sleep: DREADD Activation in Wild-Type Rats

**DOI:** 10.3390/cells11142140

**Published:** 2022-07-07

**Authors:** Andrey Kostin, Md. Aftab Alam, Anton Saevskiy, Dennis McGinty, Md. Noor Alam

**Affiliations:** 1 Research Service (151A3), Veterans Affairs Greater Los Angeles Healthcare System, 16111 Plummer Street, Sepulveda, Los Angeles, CA 91343, USA; andrey.kostin@usa.com (A.K.); aftabalam@ucla.edu (M.A.A.); dmcginty@ucla.edu (D.M.); 2 Department of Psychiatry, University of California, Los Angeles, CA 90095, USA; 3 Scientific Research and Technology Center for Neurotechnology, Southern Federal University, 344006 Rostov-on-Don, Russia; saevskiy@sfedu.ru; 4 Department of Psychology, University of California, Los Angeles, CA 90095, USA; 5 Department of Medicine, David Geffen School of Medicine, University of California, Los Angeles, CA 90095, USA

**Keywords:** sleep, hypothalamus, ventrolateral preoptic area, designer receptors exclusively activated by designer drugs, clozapine-N-oxide, Fischer-344 rats

## Abstract

The ventrolateral preoptic area (VLPO) predominantly contains sleep-active neurons and is involved in sleep regulation. The perifornical-hypothalamic area (PF-HA) is a wake-regulatory region and predominantly contains wake-active neurons. VLPO GABAergic/galaninergic neurons project to the PF-HA. Previously, the specific contribution of VLPO neurons projecting to the PF-HA (VLPO > PF-HA^PRJ^) in sleep regulation in rats could not be investigated due to the lack of tools that could selectively target these neurons. We determined the contribution of VLPO > PF-HA^PRJ^ neurons in sleep regulation by selectively activating them using designer receptors exclusively activated by designer drugs (DREADDs) in wild-type Fischer-344 rats. We used a combination of two viral vectors to retrogradely deliver the Cre-recombinase gene, specifically, in VLPO > PF-HA neurons, and further express hM3Dq in those neurons to selectively activate them for delineating their specific contributions to sleep–wake functions. Compared to the control, in DREADD rats, clozapine-N-oxide (CNO) significantly increased fos-expression, a marker of neuronal activation, in VLPO > PF-HA^PRJ^ neurons (2% vs. 20%, *p* < 0.01) during the dark phase. CNO treatment also increased nonREM sleep (27% vs. 40%, *p* < 0.01) during the first 3 h of the dark phase, when rats are typically awake, and after exposure to the novel environment (55% vs. 65%; *p* < 0.01), which induces acute arousal during the light phase. These results support a hypothesis that VLPO > PF-HA^PRJ^ neurons constitute a critical component of the hypothalamic sleep–wake regulatory circuitry and promote sleep by suppressing wake-active PF-HA neurons.

## 1. Introduction

Multiple lines of studies support that the ventrolateral preoptic area (VLPO) is critically involved in sleep regulation [1,2,3,4]. VLPO lesions result in significant sleep fragmentation and decreased total sleep amount [5]. The VLPO contains sleep-active neurons or neurons that exhibit increasing discharge from the waking to nonREM sleep transition to stable nonREM/REM sleep [6]. The evidence further suggests that these neurons are not only involved in the initiation of sleep, but rather are also involved in the building up and dissipation of homeostatic sleep drive during sleep deprivation and recovery sleep, respectively [7]. VLPO neurons also exhibit sleep-associated fos-expression (Fos-IR), a marker of neuronal activation [8,9,10]. While VLPO contains a higher concentration of sleep-active neurons, such neurons are diffusedly distributed in the POA region, including a higher concentration in another POA sub-region, the median preoptic nucleus (MnPO) [10,11]. Most of the sleep-active neurons in the VLPO contain the inhibitory neurotransmitters GABA and or galanin [12,13]. However, GABA and galanin neuronal populations are heterogeneous. The majority of the GABAergic and some galaninergic neurons in the VLPO are not sleep-active [12,13]. Even within the VLPO core, while about 80% of the neurons expressing sleep-associated Fos-IR co-express galanin mRNA, only ~50% of the galanin-expressing neurons are sleep-active in rats, the animal model used in this study [14]. Further, only ~22% of the galaninergic cells in the extended VLPO, the dorsal and medial extension of the VLPO core, are sleep-active [2,14]. Several neuro-peptides such as cholecystokinin, corticotropin-releasing hormone, and tachykinin 1 are co-expressed with VLPO GABAergic neurons, and are suggested to be involved in sleep regulation as well [15]. 

The perifornical-hypothalamic area (PF-HA), on the other hand, is primarily involved in wake-regulation and contains neurons that are active during waking and quiescent during nonREM and REM [16,17,18]. VLPO GABAergic/galaninergic neurons constitute a major source of afferents to the critical wake-regulatory systems in the hypothalamus, including the PF-HA, containing hypocretin/orexin (HCRT) and glutamatergic neurons,; the tuberomammillary nucleus (TMN), containing histaminergic neurons; and brainstem monoaminergic neurons [2,9,12,19,20,21,22,23]. Generally, reciprocal interactions between sleep-active POA and wake-active neuronal groups have been proposed to underlie sleep–wake control [1,2,24]. Recently, using transgenic mice models, the sleep–wake profiles of specific neuronal phenotypes and their contributions to sleep–wake regulation have also been reported [15,20,25,26]. Although, depending on the experimental paradigm, some inconsistencies were observed. These studies generally observed that selective chemoactivation of GABAergic or photo-stimulation of galaninergic neurons increased nonREM sleep. These studies also suggested that while most GABAergic or galaninergic neurons in the POA are not sleep-active, VLPO GABAergic/galaninergic neurons projecting to the wake-promoting systems are mostly sleep-active [20,21,22,25,27]. 

In rats, about 80% of VLPO neurons projecting to the PF-HA and TMN are inhibitory and express GABA and/or galanin [13,14]. Evidence also suggests that activation of POA neurons, including those in the VLPO, inhibits wake-active neurons in the PF-HA, including HCRT neurons [28,29]. However, previously, the specific contribution of VLPO neurons projecting to the PF-HA (VLPO > PF-HA^PRJ^) in sleep regulation in rats could not be investigated due to the lack of tools needed for specifically targeting these VLPO > PF-HA^PRJ^ neurons. Although, in recent years, because of the availability of transgenic models, mice are being increasingly used in sleep research, rats remain an attractive animal model because they seem to model many human behavioral and neuropsychiatric disorders that also affect sleep better than mice, as well as for other practical/technical reasons [30].

In this study, we determined the specific contribution of the VLPO > PF-HA^PRJ^ neurons in sleep regulation by selectively activating them using designer receptors exclusively activated by designer drugs (DREADDs) in wild-type (WT) Fischer-344 rats. The rats were unilaterally injected with AAV2retro-EF1a-Cre into the PF-HA to deliver Cre-recombinase into VLPO > PF-HA^PRJ^ neurons retrogradely, and AAV-hSyn-DIO-hM3D(Gq) was injected ipsilaterally into the VLPO for the Cre-dependent selective expression of hM3Dq receptors, a modified excitatory human muscarinic M3 receptor, in those neurons. We found that the selective activation of VLPO > PF-HA^PRJ^ neurons promotes nonREM sleep during the early dark phase when rats are typically awake, and sleep propensity is the lowest, and after exposure to the novel environment, which induces acute arousal and is used as an animal model of insomnia during the light phase. 

## 2. Materials and Methods

### 2.1. Experimental Subjects

The experiments were conducted on 14 young (4–5 months old) male Fischer-344 rats; however, the data from one rat could not be used due to mechanical failure. These rats were maintained on a 12 h: 12 h light: dark cycle (lights on at 9:00 h), an ambient temperature of 24 ± 2 °C, and with food and water available *ad libitum*. All experiments were conducted in accordance with the National Research Council’s “Guide for the Care and Use of Laboratory Animals” and approved by the Institutional Animal Care and Use Committee of the Veterans Affairs Greater Los Angeles Healthcare System; Protocol # 1615739.

### 2.2. Surgical Procedures

The details of the surgical procedures have been described previously [31]. Briefly, under surgical anesthesia (100 mg/Kg ketamine + 15 mg/Kg xylazine; maintenance with isoflurane) and under aseptic conditions, two holes (~1 mm diameter), one above the VLPO and another above the PF-HA, were drilled ipsilaterally into the skull. The viral vectors were injected unilaterally into the VLPO (AP: −0.5 mm, LM: 1.0 mm, and DV: 9.2 mm, relative to bregma) and into the PF-HA (AP: −3.0 mm, LM: 1.4 mm, and DV: 9.0 mm) [32] using a micro-syringe with 28 gauge needle (Hamilton Co., Reno, NV, USA) and attached to a motor-driven micromanipulator. Electroencephalogram (EEG) and electromyogram (EMG) electrodes were implanted stereotaxically for polygraphic monitoring of sleep–waking states. Stainless steel screw EEG electrodes were implanted through small, hand-drilled holes in the skull, and flexible silver stainless steel EMG wire electrodes were implanted into the dorsal cervical musculature. 

### 2.3. Experimental Groups and Viral Vectors

**Figure 1:**
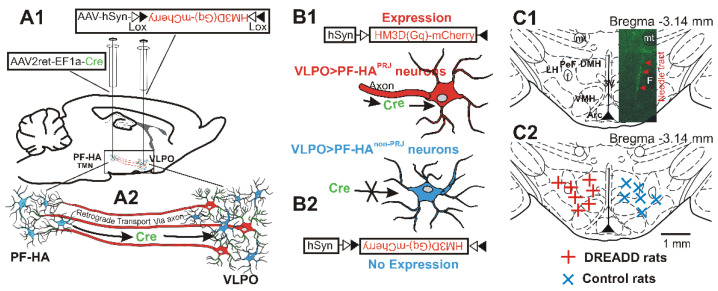
**Schematic of the procedure used for the chemoactivation of VLPO > PF-HA^PRJ^ neurons**. (**A1**,**A2**): schematic representation of viral vector injections, AAV2retro-EF1a-Cre in the PF-HA, and AAV-hSyn-DIO-hM3D(Gq) in the VLPO, and the retrograde transport of Cre-recombinase into the subset of VLPO neurons projecting to the PF-HA (**A2**). (**B1**,**B2**): Cre-dependent expression of hM3Dq-mCherry in VLPO > PF-HA^PRJ^ neurons (**B1**) and not in the VLPO neurons not projecting to the PF-LH (VLPO > PF-HA^non-PRJ^) neurons (**B2**). This approach was used to specially target and examine the role of VLPO > PF-HA^PRJ^ neurons and their interactions with the PF-HA neuronal groups in sleep–wake regulation. (**C1**,**C2**): AAV2retro-EF1a-Cre injection sites in the PF-HA. Although the AP locations of the injection sites varied by 100–200 µm, these are shown in one plane (Bregma −3.14 mm; adapted with permission from Paxinos and Watson, 1998 [32]). (**C1**): photomicrograph of a representative histological section showing the needle tract, as indicated by the increased autofluorescence, which was used to identify injection sites in the PF-HA. (**C2**): The locations of all injections in the DREADD and non-DREADD control groups. The locations of injection sites were broadly comparable in both the DREADD and control groups. Thus, it is likely that similar groups of VLPO > PF-HA^PRJ^ neurons were retrogradely labeled in both DREADD and control groups. 3V, third ventricle; Arc, arcuate nucleus; DMH, dorsomedial hypothalamus; f, fornix; LH, lateral hypothalamus; mt, mammillothalamic tract; PeF, perifornical nucleus; TMN, tuberomammillary nucleus; VLPO, ventrolateral preoptic area; VMH, ventromedial hypothalamus.

***DREADDs group****:* We used AAV2retro with the gene of Cre-recombinase, already validated to propagate retrogradely, to create the genetic switch to turn on recombinant dependent effector and reporter genes in VLPO > PF-HA^PRJ^ neurons and then further express the double-floxed hM3Dq gene in them for their activation by CNO [33,34,35]. This group of rats was injected with AAV2retro-EF1a-Cre (Addgene #55636; volume, 1.0 µL; titer of vector, 3.5 × 10¹² vg/mL) into the PF-HA and Syn-driven, Cre-dependent, hM3D(Gq) receptor genes with an mCherry reporter, AAV2-hSyn-DIO-hM3D(Gq)-mCherry (Addgene #44361, volume 0.4 µL; titer of vector, 3.5 × 10¹² vg/mL) into the VLPO. The vectors were injected at 0.1 µL/2 min. After completion of the injection, the needle was left in place for another 20 min and then slowly withdrawn to avoid any backflow. AAV2-hSyn-DIO-hM3D(Gq)-mCherry injections into the VLPO resulted in Cre-dependent hM3Dq expression (as evident from the mCherry labeling) exclusively in the VLPO > PF-HA^PRJ^ neurons (Figure 1)

***Control group:*** Using the same protocol as in the DREADDs group, this group of rats was also injected with the same AAV2retro-EF1a-Cre into the PF-HA for delivering Cre-recombinase into VLPO > PF-HA^PRJ^ neurons retrogradely. As a control, we injected AAV2-DIO-GCaMP6s-dTomato, available on hand, into the VLPO, resulting in Cre-dependent GCaMP expression as a reporter (as evident from the dTomato labeling) exclusively in the VLPO > PF-HA^PRJ^ neurons. Unlike hM3Dq in the DREADD group, the GCaMP6 protein created by the synthetic fusion of GFP, calmodulin, and the additional peptide could not be activated by CNO [36]. 

### 2.4. Recovery and Adaptation

Rats were allowed to recover from the surgical procedure for 10–14 days in Plexiglas recording cages placed in a sound-attenuated and temperature-controlled recording chamber. The rats were then connected with the recording cable and allowed an additional seven days for acclimatization to the recording setup and recording cables before the experiments were carried out. During this time, the rats were also adapted to the handling, similar to that they were subjected to during the injection procedure. In the recording chamber, the rats were maintained at the same 12 h: 12 h light: dark cycle, with *ad libitum* access to food and water. 

### 2.5. Data Acquisition

The actual experiment was started after ~4 weeks of surgical procedure/viral vector injections, allowing sufficient time for the retrograde labeling and the expression of hM3Dq in VLPO > PF-HA^PRJ^ neurons. The amplified and filtered EEG and EMG signals were continuously digitized and stored on the computer’s hard disk using an integrated computer interface device (Cambridge Electronic Design 1401, UK; supporting software, Spike 2) for subsequent sleep–wake scoring and analyses. To determine the specific contributions of the VLPO > PF-HA^PRJ^ neurons in sleep regulation, we chemo-activated VLPO > PF-HA^PRJ^ neurons by CNO. We conducted the following two sets of studies:

***Experiment 1:*** This study was conducted during the first half of the dark phase, when rats are largely awake and sleep propensity is the lowest. The DREADDs and the control group of rats were injected SQ with saline or CNO at dark-onset, and their EEG and EMG were continuously recorded in their home cages without any disturbance for 6 h. For SQ injection, the rats were gently taken out of the cage without disconnecting the recording cable and placed on a plastic platform (25 × 25 cm) near the cage. The skin flap around the lumbar part of the body and from the backside was gently pulled up, and the CNO was injected. After injection, the rats were gently returned to their cages. Saline or CNO was injected (200 nL) using an insulin syringe (needle 27 G), and between injections, each rat had a break of 2–3 days in the case of CNO and 1–2 days in the case of the saline treatments. The injection of saline and various doses of CNO were randomized. The injection of each rat took about 1 min. We chose SQ injection instead of IP, which has typically been used in other studies because it is faster, because rats exhibit much less behavioral stress and vocalization during SQ injection. Although we did not assess the difference between the latency of the effects after IP and SQ injections, the SQ route likely has longer latency. We started with a dose of 0.3 mg/kg of CNO and further determined the effects of increasing its amounts to 1.0 mg/kg and 3.0 mg/kg.

***Experiment 2:*** In rodents, acute exposure to the novel environment (e.g., bedding changes/new cage) suppresses sleep and causes arousal for 1–3 h [20,37,38]. We determined whether chemoactivation of the VLPO > PF-HA^PRJ^ neurons helps improve sleep in this model of insomnia. Both the DREADDs and the control group of rats were injected with either saline or CNO (1 mg/kg) 6 h after lights-on. After 20 min of injection, the rats were transferred to a new cage, similar to the home cage, with fresh/new bedding and nesting materials, and their EEG and EMG were continuously recorded for 5–6 h or until the onset of the dark phase. We chose the middle of the light phase so that the animals would have less sleep pressure and were more prone to novel-environment-induced arousal. In addition, animals were exposed to the novel environment 20 min after saline or CNO injections so that the CNO-induced activation of the VLPO > PF-HA^PRJ^ neurons coincided with the exposure to the novel environment. 

To quantify the population of neurons that were activated by CNO, at the end of the experiment, 1.0 mg/kg of CNO was injected using the same experimental paradigm, and after 90 min, the rats were perfused and their brains were harvested for immunohistochemical processing. 

### 2.6. Histology

At the end of the experiment, under deep anesthesia (100 mg/kg, IP, pentobarbital, Vortech, Dearborn, MI, USA), the rats were perfused transcardially with 40 mL of 0.1 M phosphate buffer saline (PBS, pH 7.2), followed by 500 mL of 4% paraformaldehyde in PBS. Their brains were removed and equilibrated in 10%, 20%, and, finally, 30% sucrose until they sank. The coronal sections from the POA to the posterior hypothalamus, including the sites of viral vector injections in the VLPO and PF-HA, were freeze-cut at a 30 μm thickness. Free-floating sections were immunostained to visualize VLPO > PF-HA^PRJ^ neurons expressing hM3Dq or GCaMP and c-fos immunoreactivity using standard protocols [8,12]. Briefly, the sections were incubated with anti-c-fos rabbit polyclonal antibody (1:2000; cat. #ABE475, EMD Millipore Corporation, Temecula, CA, USA) for 72 h at 4 °C, and then developed with secondary antibody (1:400, horse anti-rabbit DaiLight 647; Vector Laboratories Inc., Newark, CA, USA). The needle tracks and injection sites in the PF-HA were identified and mapped. 

### 2.7. Data Analyses

***Sleep–wake parameters:*** The sleep–wake quantity of the animals was scored manually in 10 s epochs in terms of waking (consisting of active and quiet-waking), nonREM sleep, and REM sleep using SleepSign software (version 2; Kissei Comtec Co., Tokyo, Japan) and standard criteria [39]. Six hours of night recording and five hours of day recordings after exposure to the novel environment were analyzed after saline and CNO injections. We found 1 mg CNO to be optimally effective in inducing sleep–wake changes. We examined the continuity and stability of the sleep–waking state after CNO treatments in the DREADDs and control group by further classifying episode duration of waking (active), nonREM sleep, and REM sleep stages into four subcategories: episodes with a duration of < 30 s (very short), > 30 s to 2 min (short), > 2 min to 5 min (medium), and > 5 min (long). The changes in sleep–wake profiles after CNO treatments were analyzed during 0–3 and 3–6 h during the dark phase and 0–2 and 2–5 h after exposure to the novel environment to determine if the effects were time-dependent. 

***Confocal imaging and cell counting:*** The number of hM3Dq+ (mCherry+), c-fos+, GFP+ (dTomato+), and hM3Dq+ or GFP+ cells expressing c-fos were counted on ipsilateral, as well as contralateral, sides in the CNO-treated DREADDs and non-DREADD control groups using an LSM 900 Zeiss confocal microscope and ImageJ software (https://imagej.net/software/fiji, accessed on 11 March 2022). Images of 2-4 sections encompassing the full extent of VLPO > PF-HA^PRJ^ neurons expressing hM3Dq, GFP, and Fos-IR were obtained using the fluorescent filters 594, 488 (VLPO > PF-HA^PRJ^ neurons), and 647 (c-fos). No mCherry+/hM3Dq+ or dTomato+/GFP+ cells were observed on the contralateral side.

***Statistical analyses:*** The SigmaPlot (Systat Software, San Jose, CA, USA) software package was used for statistical analyses of the data. The data are presented as mean ± standard error of the mean (SEM). One-way repeated-measures analysis of variance (ANOVA) for within-group analysis and one-way ANOVA for between-group analysis, followed by multiple pairwise comparisons using the Holm–Sidak test, were used for comparing sleep–wake changes. In a few cases, the data failed normality or equal variance tests. Those data points were analyzed by both parametric and equivalent non-parametric methods (Friedman RM ANOVA on ranks or Kruskal–Wallis one-way ANOVA on ranks) and the more conservative of the two levels of significance was used. The sleep–wake changes or changes in Fos-IR in GFP+ or mCherry+ cells after CNO treatments between the DREADD and non-DREADD groups were compared using the Student’s *t*-test or Mann–Whitney rank sum test in the case where the normality test failed. 

## 3. Results

### 3.1. Labeling of VLPO > PF-HA^PRJ^ Neurons and hM3Dq Receptor Expression

The locations of the microinjection sites of the AAV2-EF1a-Cre in the PF-HA and the distribution of the retrogradely labeled VLPO > PF-HA^PRJ^ neurons along with hM3Dq or GFP expression are shown in Figure 1 and Figure 6. The AAV2retro-EF1a-Cre vector did not produce fluorescent tags; therefore, the locations of its injection sites were confirmed by the needle tracts (Figure 1C). 

Although the locations of the AAV2retro-EF1a-Cre injection sites varied rostrocaudally and dorsoventrally by 100–200 µm, all of them were localized in the perifornical area, in portions of the dorsomedial and ventromedial hypothalamus and the lateral hypothalamus. The extent of the parenchymal propagation of AAV2retro is similar to AAV2, which has minimal propagating capacity compared to other serotypes and is estimated to be ≤ 1 mm from the injection site [40,41,42]. Thus, it is likely that in this study, AAV2retro-EF1a-Cre diffused into ~1 mm area from the point of injections including the HCRT and glutamatergic neuronal fields (Figure 1C). In the VLPO, the accuracy of both VLPO and PF-HA injections was confirmed by the Cre-dependent expression of mCherry or dTomato, which were visible only in neurons projecting to the PF-HA from where the Cre-recombinase was retrogradely transported to VLPO > PF-HA^PRJ^ neurons (Figure 1 and Figure 6). In the VLPO, we observed a relatively higher density of hM3Dq or GCaMP6 labeled neurons in the VLPO core and, to some extent, in the medial POA and dorsolateral POA, indicating that the sleep–wake changes observed in this study were predominantly due to the activation of VLPO > PF-HA^PRJ^ neurons localized in the VLPO core and, to a lesser extent, in the extended VLPO. 

### 3.2. Chemoactivation of VLPO > PF-HA^PRJ^ Neurons Causes Sleep during the Dark Phase

**Figure 2:**
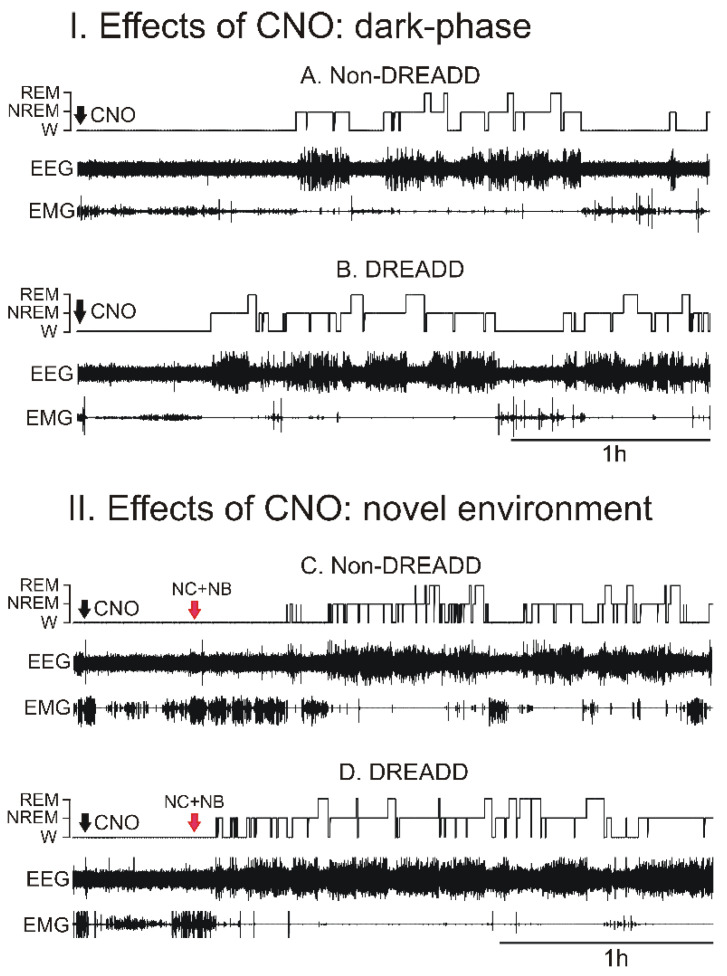
**Effects of the chemoactivation of VLPO > PF-HA^PRJ^ neurons on sleep-waking during the dark phase and after exposure to a novel environment during the light phase**. Examples of continuous EEG and EMG tracings showing the sleep–wake profiles of animals after 1.0 mg/kg CNO treatments (black arrowhead) in the non-DREADD control and the DREADD group of rats during the dark phase and after placing them in a new cage with new bedding materials (red arrowhead). The CNO-treated DREADD rats entered into sleep relatively quickly and spent more time sleeping, both during the dark phase and the after cage and bedding change. NC + NB, new cage + new bedding material.

**Figure 3:**
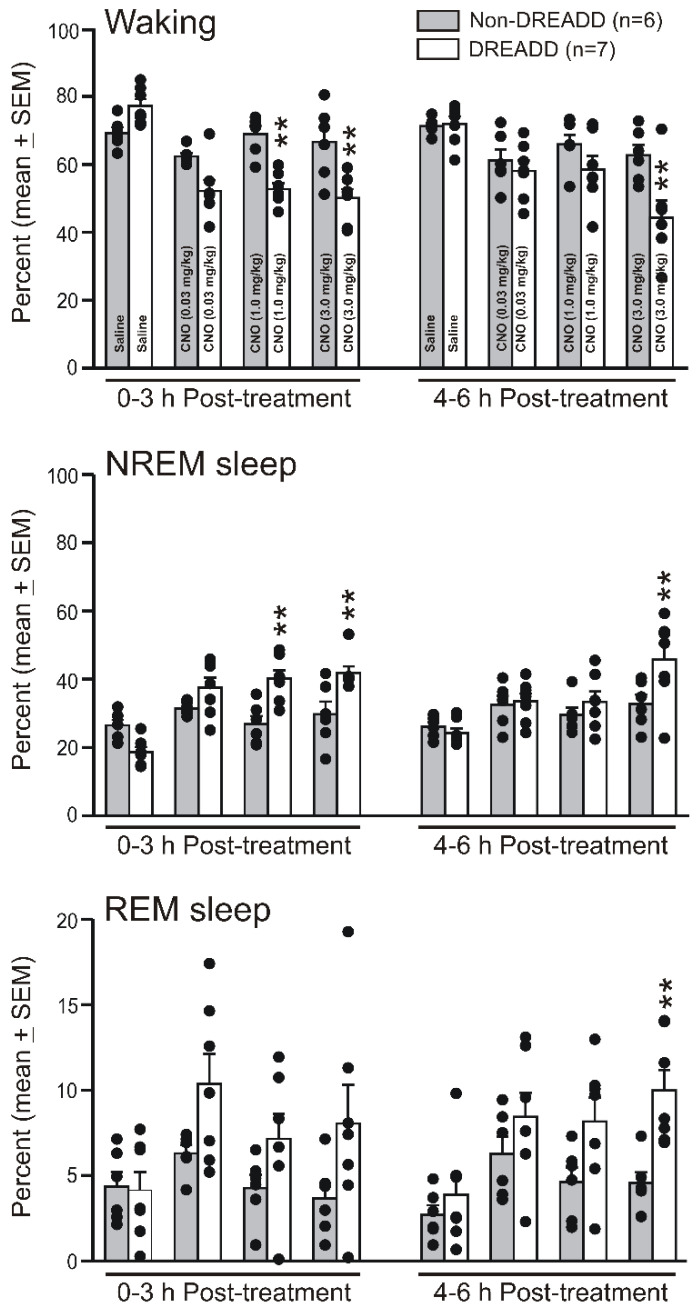
**Effects of the chemoactivation of VLPO > PF-HA^PRJ^ neurons on sleep–waking during the dark phase.** The percent of time spent by the individual non-DREADD and DREADD rats (filled circles) and as respective groups (mean ± SEM; bars) in waking, nonREM sleep, and REM sleep during the six h (divided into first three and last three h) of recording after saline and three doses (0.3, 1.0, and 3.0 mg/kg) of CNO treatments. The treatments are indicated in the columns. The CNO-treated DREADD rats spent significantly less time in waking and more time in nonREM and, to some extent, REM sleep. The effects were time-dependent and more pronounced during the first three h after the CNO injection. Except for the 3.0 mg/kg of CNO, the sleep–wake profiles of the DREADD and non-DREADD groups after the various treatments during 3–6 h were comparable. ** *p* < 0.01 of significance compared to the non-DREADD control (one-way ANOVA followed by Holm–Sidak pairwise multiple comparisons).

The amounts of waking, nonREM sleep, and REM sleep during 0–3 and 3–6 h after the saline and CNO injections at dark-onset in the rats expressing hM3Dq (DREADD rats) or GFP (non-DREADD rats) in the VLPO > PF-HA^PRJ^ neurons are shown in Figure 2 and Figure 3. 

*Sleep–wake changes during 0–3 h post-treatment:* In the non-DREADD rats, the effects of various doses of CNO on waking, nonREM sleep, and REM sleep during the 0–3 h post-injection recording during the dark phase were comparable and similar to that observed after the saline injection (one-way RM ANOVA; F_5,3_ = 1.61–2.29; *p* > 0.05). However, in the DREADD rats, CNO treatment significantly decreased the amount of waking (one-way RM ANOVA, F_6,3_ = 34.35, *p* < 0.001) and increased the amount of nonREM sleep (F_6,3_ = 38.89, *p* < 0.001) during the first three h of the post-injection period. CNO also caused a marginal increase in REM sleep (overall, F_6,3_ = 6.26, *p* < 0.01; significant difference only between saline and 0.3 mg/kg CNO; Holm–Sidak multiple comparisons).

The spontaneous sleep–waking profiles of both the non-DREADD and DREADD rats during the 0–3 h recording after saline injections were comparable. However, compared to non-DREADD group, the effects of CNO treatment on waking (F_7,44_ = 15.40; *p* < 0.001) and nonREM sleep (F_7,44_ = 12.07; *p* < 0.001) in the DREADD rats were significant (Figure 2 and Figure 3). The DREADD group exhibited only a marginal increase in REM sleep (*p* = 0.03; Kruskal–Wallis one-way ANOVA on ranks, no intergroup differences). Among the CNO doses, 1 mg/kg was the minimal dose that produced consistent effects on sleep-waking. While CNO at a 3 mg/kg dose induced a stronger response on sleep during 0–3 h, it was not significantly different from the other doses used in this study (Figure 3). 

**Figure 4:**
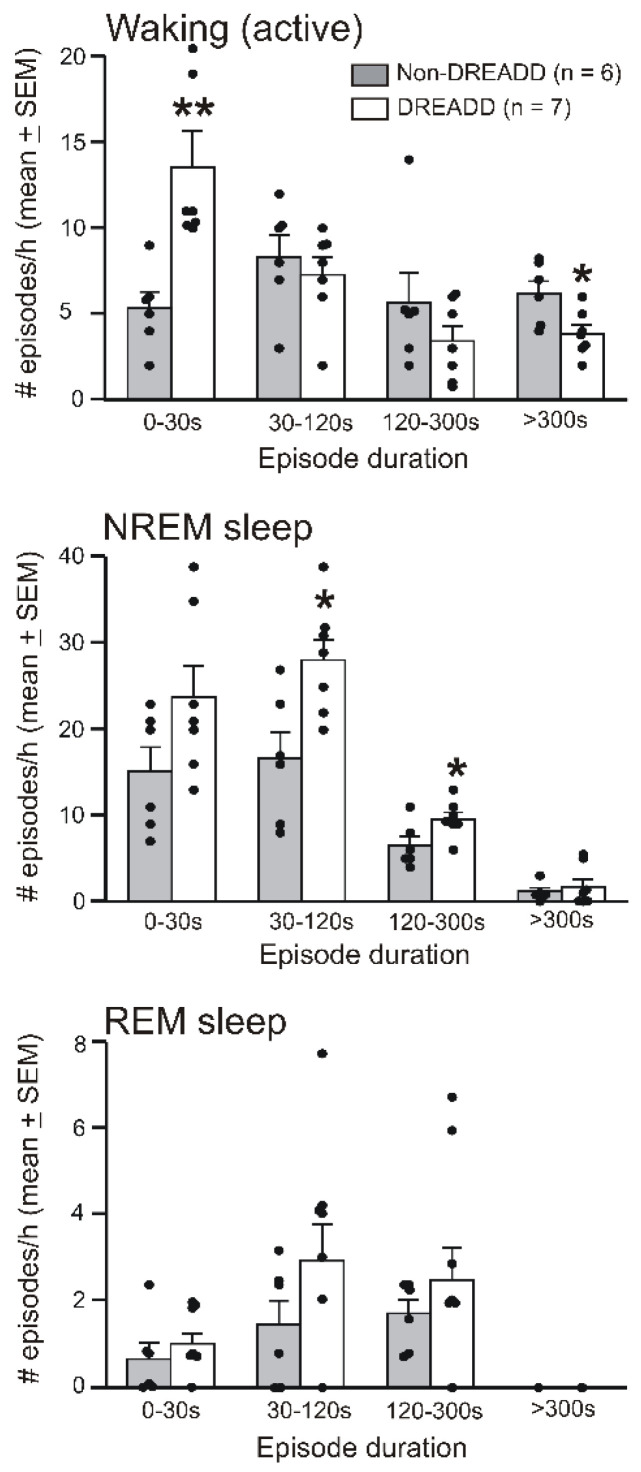
**Effects of the chemoactivation of VLPO > PF-HA^PRJ^ neurons on sleep–waking stability.** The numbers of episodes of waking, nonREM sleep, and REM sleep of various durations exhibited by individual rats (filled circles) and as a group (mean ± SEM; bars) during the first 3 h after 1 mg/kg of CNO treatment in the non-DREADD and DREADD rats. Compared to the non-DREAD rats, the DREADD rats exhibited a decline in the number of longer bouts of waking, although the frequency of very short episodes of waking increased significantly. These rats also exhibited significant increases in short and medium bouts of nonREM sleep. REM sleep episodes were largely not affected. * *p* < 0.05; ** *p* < 0.01; independent *t*-test.

Since CNO-induced sleep–wake changes occurred predominantly during the first 3 h, we further analyzed the extent of sleep–waking stability during 0–3 h after CNO treatment in the DREADD and non-DREADD rats. We counted the frequency of waking (active), nonREM sleep, and REM sleep episodes of very short (≤30 s), short (>30 s–2 min), medium (>2 min–5 min), and longer (>5 min) durations after 1 mg/kg of CNO treatments in the non-DREAD and DREAD rats, as described earlier [43]. Compared to the non-DREADD rats, the CNO-treated DREADD rats exhibited: (a) significantly decreased numbers of longer bouts (t = 2.61; *p* < 0.05) and an increased number of very short bouts of active-waking (t = 5.11; *p* < 0.01); and (b) significant increases in the number of short (t = 2.97; *p* < 0.05) and medium bouts of nonREM sleep (t = 2.34; *p* < 0.05). Although the frequency of nonREM sleep episodes of < 30 s duration was also higher, it was not significant (t = 1.83; *p* = 0.09). There was no significant change in the frequency of REM sleep episodes of any bout duration, although the number of small and medium bouts of REM sleep was relatively higher (Figure 4). 

*Sleep–wake changes during 3–6 h post-treatment:* During 3–6 h of post-treatment recording, in the non-DREADD rats, the effects of the various doses of CNO on waking, nonREM sleep, and REM sleep were comparable to that observed after the saline injection. In the DREADD rats, compared to the saline control, the effects of CNO on sleep–waking states remained significant (waking; one-way RM ANOVA, F_7,44_ = 7.13, *p* < 0.001; nonREM sleep, F_7,44_ = 5.72, *p* < 0.001; REM sleep, F_7,44_ = 5.34, *p* < 0.001) during the 3–6 h post-treatment period. However, compared to the non-DREADD rats, these effects were insignificant after the saline and 0.3 and 1.0 mg/kg injections of CNO. Only the rats treated with 3.0 mg/kg of CNO exhibited significantly decreased waking and increases in nonREM and REM sleep during the 3–6 h post-treatment period, suggesting that the CNO effect was dose-dependent (Figure 3).

### 3.3. Chemoactivation of VLPO > PF-HA^PRJ^ Neurons Increases Sleep in an Animal Model of Insomnia

Evidence supports that after acute exposure to a novel environment, rodents are not able to sleep well for 1–3 h [20,37,38]. We determined if the chemoactivation of VLPO > PF-HA^PRJ^ neurons helps improve sleep during rats’ exposure to a novel environment. Both the non-DREADD and DREADD rats were injected with either saline or CNO at 1 mg/Kg, a dose that was optimally effective in causing sleep–wake changes in experiment 1. After the injections, these rats were transferred to new cages similar to their home cage, but with new bedding and nesting materials, and their sleep–wake profiles were recorded for 5 h.

**Figure 5:**
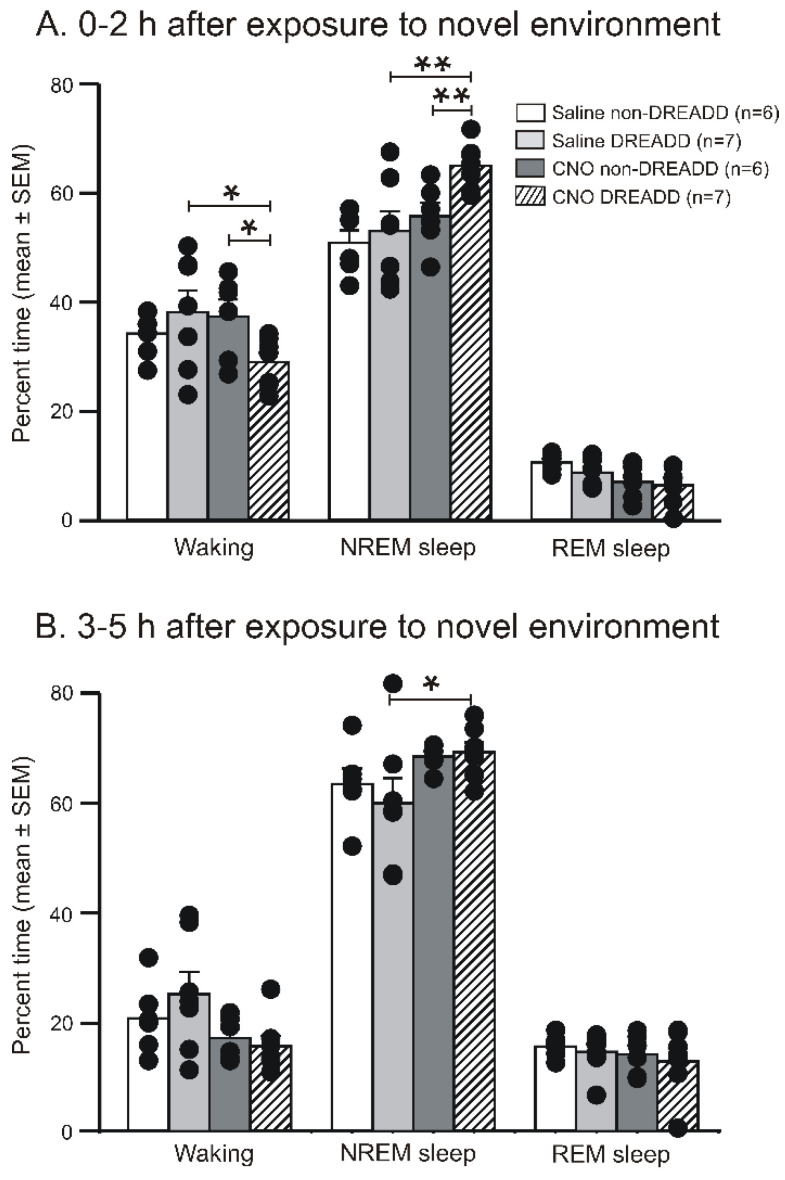
**Effects of the chemo-activation of VLPO > PF-HA^PRJ^ neurons on sleep–waking after exposure to the novel environment during the light phase.** The percent of time spent by the individual non-DREADD and DREADD rats (filled circles) and as respective groups (mean ± SEM; bars) in waking, nonREM sleep, and REM sleep during the five h (divided into first two, (**A**), and last three h, (**B**)) of recording after exposure to the novel environment plus saline and 1 mg/kg of CNO treatments. The CNO-treated DREADD rats spent significantly less time in waking and more time in nonREM sleep after exposure to the novel environment than the non-DREADD and saline control groups. * *p* < 0.05; ** *p* < 0.01 of significance (paired *t*-test for within-group and *t*-test for between-groups comparisons).

The sleep–wake profiles of the DREADD and non-DREADD rats after their exposure to a novel environment, plus the saline and CNO treatments, are shown in Figure 2 and Figure 5. The effects of saline on waking, nonREM sleep, and REM sleep in the DREADD and non-DREADD rats were comparable during the first two h of the post-injection period. However, compared to the non-DREADD rats or the saline control, the DREADD rats treated with CNO exhibited significantly reduced amounts of waking (37% versus 29%; t = 2.92; *p* < 0.05) and increased amounts of nonREM sleep (55% versus 65%; t = 3.37; *p* < 0.01) during the first 2 h of the post-injection period (Figure 2 and Figure 5A). REM sleep amounts changed marginally. The sleep–wake profiles of both groups of rats during the 2–5 h post-injection period were largely comparable (Figure 5B). 

### 3.4. Chemoactivation Increases Fos-IR in VLPO > PF-HA^PRJ^ Neurons

**Figure 6:**
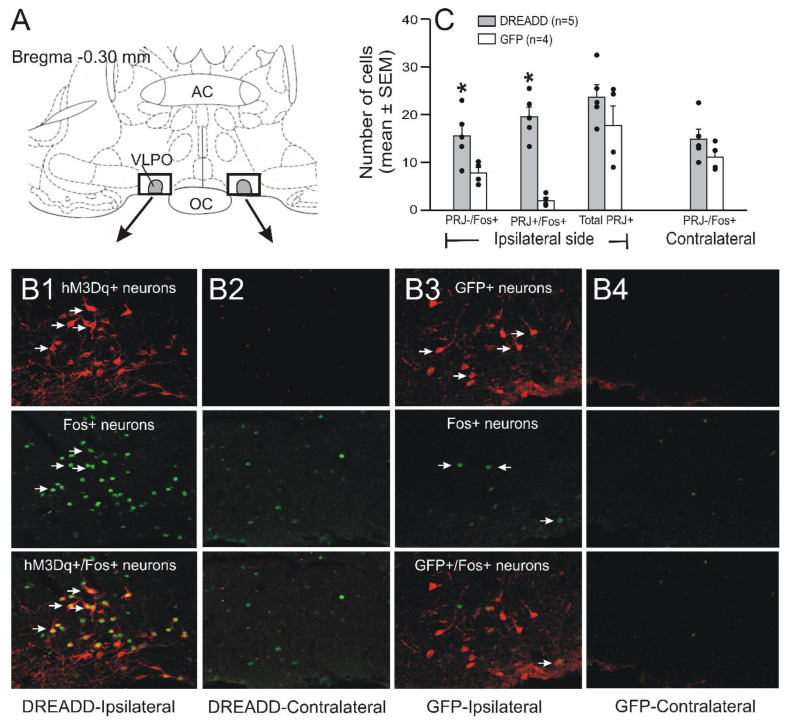
**CNO activated VLPO > PF-HA^PRJ^ neurons expressing hM3Dq**. (**A**): schematic representation of the target site and where most of the VLPO > PF-HA^PRJ^ neurons expressing hM3Dq or GFP were localized. (**B1**–**B4**): photomicrographs of representative ipsilateral and contralateral histological sections showing Fos-IR in VLPO > PF-HA^PRJ^ neurons (marked by arrows) expressing hM3Dq (mCherry; (**B1**,**B2**)) or GFP (dTomato; (**B3**,**B4**)) after 1 mg/kg of CNO injection during the dark phase. In all figures, both mCherry and GFP are represented in red, for convenience, and c-fos in green (the original staining of Fos-IR was in far-red). (**C**): The number of VLPO > PF-HA^PRJ^ and VLPO > PF-HA ^non-PRJ^ neurons expressing Fos-IR in individual rats (filled circles) and as a group (mean ± SEM; bars) after CNO treatment in the DREADD and non-DREADD rats. While the number of VLPO > PF-HA^PRJ^ neurons expressing hM3Dq or GFP was comparable, CNO significantly increased Fos-IR in VLPO > PF-HA^PRJ^ neurons expressing hM3Dq. Arrow; cell type; AC, anterior commissure; OC, optic chiasm; PRJ−, VLPO > PF-HA^non-PRJ^ neurons; PRJ+, VLPO > PF-HA^PRJ^ neurons; PRJ+/Fos+, VLPO > PF-HA^PRJ^ neurons expressing Fos-IR; PRJ−/Fos+, VLPO > PF-HA^non-PRJ^ neurons expressing Fos-IR; VLPO, ventrolateral preoptic area. * *p* < 0.05 level of significance (Mann–Whitney rank sum test).

To determine if the sleep–wake changes observed after CNO treatment in the DREADD rats during the dark phase were due to the activation of VLPO > PF-HA^PRJ^ neurons in a subset of rats, we examined the effects of 1 mg/kg of CNO on Fos-IR in VLPO > PF-HA^PRJ^ neurons in the DREADD and non-DREADD rats. The effects of CNO on Fos-IR in VLPO > PF-HA^PRJ^ neurons and VLPO neurons not projecting to the PF-HA (VLPO^non-PRJ^) in these two groups of rats during the dark phase are shown in Figure 6.

The number of labeled VLPO > PF-HA^PRJ^ neurons in the DREADD and non-DREADD rats in the POA region were comparable (*p* > 0.05; Figure 6(B1,B3 and C)). No labeled VLPO > PF-HA^PRJ^ neurons were observed in the VLPO or extended VLPO regions on the contralateral side in both groups of rats (Figure 6(B2,B4)). In the non-DREADD rats that were mostly awake after CNO treatment, fewer VLPO > PF-HA^PRJ^ and VLPO^non-PRJ^ neurons exhibited Fos-IR on the ipsilateral side (Figure 6B,C). However, in the CNO-treated DREADD rats, the number of VLPO > PF-HA^PRJ^ and VLPO > PF-HA^non-PRJ^ neurons exhibiting Fos-IR was significantly higher than the ipsilateral side of the CNO-treated non-DREADD rats (Figure 6B,C). Although the number of VLPO > PF-HA^PRJ^+/Fos+ neurons was highest in the VLPO core, VLPO > PF-HA^PRJ^ and VLPO^non-PRJ^ neurons expressing Fos-IR were also observed in its adjacent regions.

The effects of CNO on Fos-IR in the DREADD rats were mainly restricted to the ipsilateral side (Figure 6B,C). In the DREADD rats, the number of total Fos-IR neurons was significantly higher on ipsilateral side than on the contralateral side (35 versus 15; *p* < 0.05, Mann–Whitney rank sum test) or those observed ipsilaterally in the CNO-treated non-DREADD rats (35 versus 18; *p* < 0.05; Mann–Whitney rank sum test). The number of Fos+ neurons in equivalent regions on the contralateral side was comparable in the DREADD and non-DREADD rats. 

## 4. Discussion

Although the role of VLPO in sleep regulation is well recognized, the relative contributions of its various neuronal phenotypes and their interactions with different wake-promoting systems across the neuraxis remain not fully understood. In this study, we determined specific contributions of VLPO neurons projecting to the wake-regulatory PF-HA in sleep regulation by selectively activating them using DREADDs in WT Fischer-344 rats. We found that the selective activation of VLPO > PF-HA^PRJ^ neurons in freely behaving rats significantly increased sleep, especially nonREM sleep, with a concomitant decrease in waking during the early dark phase, a time when rats are typically awake and sleep propensity is minimal. The activation of VLPO > PF-HA^PRJ^ neurons also induced sleep during exposure to the novel environment during the light phase, which caused acute arousal and has been used as a rodent model of insomnia. These findings suggest that VLPO > PF-HA^PRJ^ neurons and their interactions with wake-regulatory neurons in the PF-HA constitute a critical component of the preoptic-hypothalamic sleep–wake regulatory circuitry. 

So far, transgenic mice models are being extensively used to dissect the role of specific cell types in regulating behavioral functions, including sleep–waking. Currently, transgenic rat models that may help address such questions are unavailable, although rats have been the animal model of choice for studying sleep and sleep disorders for decades. To our knowledge, this is the first study in WT rats where combinations of two viral vectors have been used to retrogradely deliver the Cre-recombinase gene specifically in VLPO > PF-HA^PRJ^ neurons and further express hM3Dq in those neurons to selectively activate them for delineating their specific contributions to sleep–wake functions. It is pertinent to note that while the AAV2-DIO- hM3D(Gq) vector injected into the VLPO likely transfected most neurons in its diffusion field, hM3Dq expression was restricted to only VLPO > PF-HA^PRJ^ neurons containing a Cre-dependent recombinant switch of this gene. In addition, hM3Dq expression in VLPO > PF-HA^PRJ^ neurons may depend on multiple factors, including efficiencies of the AAVret-vector transfection in terminals, the retrograde transportation via axons, expression of Cre and hM3D(Gq) genes, “unlocking” of hM3D(Gq) gene via recombination, and, finally, expression of the hM3D(Gq) receptors. The strength of this approach is that we were able to precisely target VLPO > PF-HA^PRJ^ neurons in WT rats and reliably study them across experimental paradigms. Such approaches, i.e., using combinations of viral vectors for targeting specific/local neuronal populations in WT animals, may also be a valuable tool in studies with limited availability of transgenic mice models or studies where maintaining transgenic models may be cost prohibitory, if available, e.g., aging research. 

The sleep–wake changes observed in this study were specifically due to the CNO-mediated activation of VLPO > PF-HA^PRJ^ neurons and not a nonspecific effect, i.e., caused by VLPO > PF-HA^nonPRJ^ neurons or CNO itself, as is evident from the following observations: (i) increased sleep with a concomitant decrease in waking after CNO injection in the DREADD group was consistent across two different physiological paradigms, reversible, and somewhat dose-dependent; (ii) various doses of CNO in the non-DREADD group did not produce any significant sleep–wake changes; (iii) saline injection in both the DREADD and non-DREADD rats did not produce any significant sleep–wake changes, and both groups exhibited comparable sleep–wake organization; (iv) CNO caused increased Fos-IR in VLPO > PF-HA^PRJ^ neurons in the DREADD group and not in the non-DREADD group, indicating that the sleep–wake changes observed after CNO injection in the DREADD group were due to the hM3Dq-mediated selective activation of VLPO > PF-HA^PRJ^ neurons; and (v) the efficacy of AAV2retro-EF1a-Cre in retrogradely delivering Cre-recombinase into projecting neurons, and that AAV-hSyn-DIO-hM3D(Gq) or AAV-DIO-GCAMP6s is selectively expressed in Cre expressing neurons, has already been validated [35,44]. However, in this study, instead of an AAV-hSyn-DIO-hM3D(Gq) control, we used AAV-DIO-GCaMP6s as a control, and we believe that it did not have any significant bearing on the outcome of this study because CNO cannot activate VLPO > PF-HA^PRJ^ neurons expressing GCaMP6s, as well as the other control findings we have described above.

In this study, CNO treatment significantly increased sleep, especially nonREM sleep, with a concomitant decrease in waking in the DREADD rats during the early dark phase, when rats are primarily awake and sleep propensity is minimal. Further, CNO treatment significantly increased c-fos expression ipsilaterally in VLPO > PF-HA^PRJ^ neurons in the DREADD animals, indicating that only the unilateral activation of VLPO > PF-HA^PRJ^ neurons contributed to the observed sleep–wake changes, and hence they were moderate. Further analysis of sleep–wake organization revealed that while the frequency of very short waking episodes increased significantly, there was a significant decline in the number of longer bouts. On the contrary, the frequency of both >30–120 s and >120–300 s episodes of nonREM sleep increased significantly. These findings are consistent with the role of VLPO sleep-active neurons in the initiation and maintenance of nonREM sleep, as their discharge activity increases during the transition from waking to sleep and remains elevated during nonREM sleep [6,7]. VLPO lesions severely shorten nonREM sleep bout duration [5]. It is plausible that while under circadian influences, the animals kept attempting to enter waking but could not maintain sustained waking due to the activation of VLPO > PF-HA^PRJ^ neurons with inhibitory influences on the wake-promoting systems in the PF-HA. This interpretation is further supported by another finding of this study that the activation of VLPO > PF-HA^PRJ^ neurons also suppressed waking and increased sleep after exposure to the novel environment, which caused acute behavioral arousal and suppression of sleep. Although we could not study the effects of CNO on Fos-IR after exposure to the novel environment, it is likely that, similar to the dark phase, sleep induced after exposure to the novel environment was also caused by the activation of VLPO > PF-HA^PRJ^ neurons by CNO. 

The DREADD animals also exhibited increased Fos-IR in VLPO > PF-HA^non-PRJ^ neurons ipsilaterally after CNO treatment. It seems unlikely that these neurons exhibited Fos-IR due to increased sleep as no such increase occurred contralaterally. It is plausible that these may be local sleep-active neurons activated by VLPO > PF-HA^PRJ^ neurons. However, it seems likely that these are VLPO > PF-HA^PRJ^ neurons with very little or no visible expression of mCherry. hM3D(Gq) and mCherry are expressed using the bi-cistronics principle. When the same promoter regulates two gene expressions, the expression of (a second) mCherry gene could be considerably weaker compared to (the first) hM3D(Gq). Thus, some neurons, while visibly mCherry-negative (single Fos), may express enough hM3D(Gq) to be activated by CNO.

In this study, we did not ascertain the neuronal phenotypes in the PF-LHA that were affected by AAV-EF1a-Cre injections and to which the VLPO > PF-HA^PRJ^ neurons projected to. However, based on the microinjection needle tips’ localization and the viral vector’s estimated diffusion field, it is likely that neurons in the PF-HA, including the perifornical area, LH, and parts of dorso- and ventro-medial hypothalamic areas, were affected. These regions predominantly contain wake-active neuronal groups, including HCRT and glutamatergic neurons, although some sleep-active neuronal groups, e.g., MCH and a subpopulation of GABAergic neurons, have also been reported [17,18,45,46,47]. The glutamatergic activation of PF-HA induces arousal and suppresses sleep in various animal models, whereas the adenosinergic inhibition of neurons in this area promotes sleep [48,49,50]. Moreover, the activation of MnPO neurons projecting to the PF-LHA predominantly inhibited wake-active neurons while exerting minimal effects on sleep-active neurons [29]. Our findings that the activation of VLPO > PF-HA^PRJ^ neurons induced sleep with a concomitant decrease in waking are consistent with these studies. 

The VLPO > PF-HA^PRJ^ neurons that were activated by CNO were localized mainly in the VLPO core. The ability of only a subset of VLPO > PF-HA^PRJ^ neurons to promote sleep, especially nonREM sleep, suggests that these neurons constitute a critical component of hypothalamic sleep-regulatory circuitry. This is consistent with findings from earlier studies that found that fos expression in the VLPO cluster neurons correlates primarily with nonREM, but not REM, sleep, and a loss of neurons in the VLPO cluster is closely related to a loss of nonREM, but not REM, sleep behavior in rats [5]. Although a significant majority of VLPO^PRJ^ neurons are GABAergic/galaninergic [2,9,12,19,20,21], the neurotransmitter phenotype(s) of VLPO > PF-HA^PRJ^ neurons examined in this study and their relative contributions or the role of co-transmitters in these neurons in influencing PF-HA neuronal groups and this circuitry remains to be fully characterized.

We had shown earlier that POA warm-sensitive neurons are predominantly sleep-active and exhibit increased sensitivity during non-REM sleep. Thus, their activation not only causes sleep, but also causes a decline in body temperature that accompanies sleep [51]. We also found that the activation of POA warm-sensitive neurons by local warming inhibits wake-active neuronal groups, including those in the PF-LHA and dorsal raphe nucleus [28,52]. Some of these warm-sensitive neurons are likely GABAergic/galaninergic VLPO^PRJ^ neurons. Thus, although we did not record body temperature, it is likely that activating VLPO > PF-HA^PRJ^ neurons would also induce hypothermia, as reported earlier after the activation of VLPO galaninergic neurons [20]. 

Further, the evidence supports the conclusion that VLPO neurons project to multiple wake-promoting regions in the hypothalamus and brainstem. The activation of VLPO neurons has been shown to inhibit PF-HA neurons and neurons in other arousal systems they project to [28,52]. It is plausible that in this study, the VLPO > PF-HA^PRJ^ neurons that were activated by CNO also projected to other wake-promoting regions; thus, the possibility that their activation caused the simultaneous inhibition of other wake-promoting systems, contributing to the evoked sleep response, cannot be ruled out. The validation of such interpretations requires further studies. In addition, the evidence suggests that gonadal hormones may affect the sleep–wake organization, as well as Fos-IR in VLPO neurons, differently in male and female subjects [53,54,55]. To avoid these confounds, we used only male animals, which created a limitation of this study. 

## 5. Conclusions

This study found that the selective activation of VLPO > PF-HA^PRJ^ neurons induces sleep, with a concomitant decrease in waking during the dark phase, when rats are typically awake, and after exposure to a novel environment during the light phase, which causes acute arousal, and has been used as a rodent model of insomnia. These findings suggest that VLPO > PF-HA^PRJ^ neurons and their interactions with wake-regulatory PF-HA neurons constitute a critical component of the preoptic-hypothalamic sleep–wake regulatory circuitry.

## Data Availability

The result section sufficiently describes the data. Further details or the raw data or analysis done are available for sharing/presentation upon request to the corresponding author.
